# Basic Motor Competencies of 6- to 8-Year-Old Primary School Children in 10 European Countries: A Cross-Sectional Study on Associations With Age, Sex, Body Mass Index, and Physical Activity

**DOI:** 10.3389/fpsyg.2022.804753

**Published:** 2022-04-25

**Authors:** Marina Wälti, Jeffrey Sallen, Manolis Adamakis, Fabienne Ennigkeit, Erin Gerlach, Christopher Heim, Boris Jidovtseff, Irene Kossyva, Jana Labudová, Dana Masaryková, Remo Mombarg, Liliane De Sousa Morgado, Benjamin Niederkofler, Maike Niehues, Marcos Onofre, Uwe Pühse, Ana Quitério, Claude Scheuer, Harald Seelig, Petr Vlček, Jaroslav Vrbas, Christian Herrmann

**Affiliations:** ^1^Department of Sport, Exercise and Health, University of Basel, Basel, Switzerland; ^2^Department of Educational Sciences & Didactics in Sport, University of Potsdam, Potsdam, Germany; ^3^School of Education, University College Cork, Cork, Ireland; ^4^Institute of Sports Sciences, Goethe University Frankfurt, Frankfurt, Germany; ^5^Institute of Human Movement Science, University of Hamburg, Hamburg, Germany; ^6^Department of Motor Sciences, University of Liège, Liège, Belgium; ^7^Department of Physical Education and Sport Science, National and Kapodistrian University of Athens, Athens, Greece; ^8^Department of School Education, Trnava University, Trnava, Slovakia; ^9^Institute for Sport Studies, Hanze University of Applied Sciences, Groningen, Netherlands; ^10^Institute of Didactics, Teaching and School Development, Salzburg University of Education Stefan Zweig, Salzburg, Austria; ^11^Centro de Estudos de Educação, Faculdade de Motricidade Humana & Unidade de Investigaçao e Desenvolvimento em Educação e Formação, Instituto de Educação, Universidade de Lisboa, Lisbon, Portugal; ^12^Department of Education and Social Work, University of Luxembourg, Luxembourg, Luxembourg; ^13^Department of Physical Education and Health Education, Masaryk University, Brno, Czechia; ^14^Physical Education Research Group, Zurich University of Teacher Education, Zurich, Switzerland

**Keywords:** motor competence, physical activity, FMS, MOBAK, motor development, screening, physical education, motor skills

## Abstract

Basic motor competencies (BMC) are a prerequisite for children to be physically active, participate in sports and thus develop a healthy, active lifestyle. The present study provides a broad screening of BMC and associations with age, sex, body mass index (BMI) and extracurricular physical activity (PA) in 10 different European countries. The different country and regional contexts within Europe will offer a novel view on already established BMC associations. The cross-sectional study was conducted in 11 regions in 10 European countries in 2018. The motor competence areas, *object movement* (OM) and *self-movement* (SM), were assessed using the MOBAK-1-2 test instrument in 3758 first and second graders (age: *M* = 6.86 ± 0.60 years; 50% girls) during Physical Education classes. Children were questioned about their extracurricular PA and age. Their body weight and height were measured in order to calculate BMI. Statistical analyses included variances and correlations. The results showed significant differences in BMC levels between countries (OM: *F* = 18.74, *p* < 0.001, η^2^ = 0.048; SM: *F* = 73.10, *p* < 0.001, η^2^ = 0.163) whereas associations between BMC and correlates were similar. Boys performed significantly better in OM while girls performed better in SM. Age was consistently positively related to OM and SM with older children reaching higher levels of BMC than younger ones. While participation rates for extracurricular PA differed widely, participation in ball sports was correlated with OM and SM. Participation in individual sports showed a significant association with SM. In summary, BMC levels of children seem to depend on where they live and are strongly related to their participation in extracurricular PA. Therefore, education and health policies, in order to enhance motor competence development and PA participation, are recommended. Further research on country-specific Physical Education frameworks and their influence on BMC will provide more insights into structural factors and cultural characteristics of BMC development. On a school level, support tools and educational materials for teachers about BMC may enable children to achieve a basic level of motor competencies through Physical Education, contributing to lifelong participation in PA.

## Introduction

Physical inactivity among children and adolescents is a global health concern. In 2018, only between 20 and 40% of 5- to 17-year-old children in Europe met the WHO recommendations of 60 min moderate to vigorous intensity physical activity (PA) daily (Aubert et al., 2018). Motor competence is directly related to PA and various health parameters such as health-related fitness and weight status (Pill and Harvey, 2019; Valentini et al., 2020). These dynamics are causal for the engagement or disengagement in PA (Stodden et al., 2008; Robinson et al., 2015; Utesch et al., 2019). In international health science research, motor competence serves as an umbrella term for common terminologies, like motor proficiency, motor performance or fundamental movement skills, to describe goal-oriented movements that include fine and gross motor skills and activities, motor coordination and whole body movements (Robinson et al., 2015; Haga et al., 2018). An additional aspect of motor competence is the concept of basic motor competencies (BMC) (Scheuer et al., 2019b). Its foundation lies in the competence concept of European educational science, where a major interest is monitoring the learning outcomes of physical education (Weinert, 2001; Klieme and Hartig, 2007). BMC are functional performance dispositions which emerge from the demands of context-specific situations (Herrmann et al., 2017). They are a precondition for developing higher motor competence levels and more sport-specific skills. Therefore, a certain level of BMC in children is essential for age-adequate sports engagement (Schierz and Thiele, 2013). BMC are distinct from general motor abilities (e.g., strength) and from concrete motor skills (e.g., handstand). BMC exert a control function over these motor performance dispositions and thus complement them (Herrmann et al., 2017, 2019; Utesch and Bardid, 2019).

Assessments of BMC relate specifically to the context of Physical Education curriculum (Herrmann and Seelig, 2017). They are grade- and age-specific and product-oriented, focusing on successful achievement of the movement goal rather than on the quality or quantity of movement execution (Bardid et al., 2019; Herrmann et al., 2019; Barnett et al., 2021; Lopes et al., 2021). This distinguishes them from measurements of motor skills, which are mostly assessed by process-oriented assessments (Logan et al., 2018), or motor abilities, which are task- and context-independent (Bös, 2016). Common BMC assessments like the MOBAK-1-2 test instrument (Herrmann et al., 2015; Herrmann, 2018) examine the two competence areas object movement (OM) and self-movement (SM). As BMC themselves are latent constructs and therefore not directly visible, the competence areas are each examined by the performance in four age-adequate manifest motor qualifications. Examples include bouncing a ball through a corridor (for OM) or running in a given sequence (for SM). The BMC concept is transferable to other dimensions and competence areas. E.g., the MOBAK-LUX test instrument consists of four competence areas, all operationalized by test items that are embedded in the Luxembourgish curriculum (Scheuer et al., 2019a).

Childhood, especially primary school ages, is a crucial period for motor development. Promoting PA and motor competence particularly at this developmental stage is highly beneficial for a healthy and active lifestyle (Goodway et al., 2019). One of the key tasks of Physical Education is therefore to provide children with basic motor competencies in order to be physically active and participate in sports, both within Physical Education settings and outside school in extracurricular activities (European Commission/EACEA/Eurydice, 2013; SHAPE America, 2014; Vlček, 2019). In order to ensure that children can practice and improve their BMC in Physical Education, it is important to be aware of their level of BMC and to provide appropriate and specific support to children and their teachers.

Children in primary school, however, differ in their BMC-levels, due to endogenous and exogenous factors. The associations of BMC with determinants like age, body mass index (BMI), sex and extracurricular PA have been investigated in various studies (Tumynaitë, 2016; Herrmann, 2018; Quitério et al., 2018; Herrmann et al., 2019; Scheuer et al., 2019a; Strotmeyer et al., 2020). Studies showed that older children perform better in BMC than younger children of the same school grade (Herrmann et al., 2017). These differences in BMC between children of the same grade are more apparent in preschool settings (Kühnis et al., 2019) and decrease across primary school (Herrmann and Seelig, 2017; Strotmeyer et al., 2020). Children with a lower BMI achieve higher BMC scores in the motor competence area SM than children with a higher BMI (Herrmann et al., 2019). While boys achieve better results in the competence area OM, girls are slightly better in SM (Herrmann, 2018). On the other hand, a recent study has shown that not sex itself, but sex-specific sports socialization (extracurricular participation in ball sports or individual sports) is a predictor of BMC (Gramespacher et al., 2020). Studies in Germany and Switzerland have shown that the correlations between extracurricular PA and certain fields of BMC were moderate to high and that BMC were a predictor for participation in extracurricular PA (Herrmann and Seelig, 2017; Herrmann et al., 2017). More precisely, children who play extracurricular ball sports have higher BMC values in OM, while children who participate in individual sports have better values in SM than children who are not active in the respective area (Herrmann et al., 2017). Scheuer and Bund (2018) found similar differences in OM and SM levels between girls and boys among school children in Luxembourg. The research group further examined the need of support (defined as failing one third or more of the test tasks in one competence area) and found that around 23% of the first and third graders needed support with OM. In addition, students with a migration background, no activity in a sports club or with overweight had a higher need of support than those without these characteristics. In sum the overall associations between age, BMI, sex and extracurricular PA with BMC are similar across the countries tested so far.

Physical Education curricula as well as approaches to sports in general are very similar across Europe, and BMC are the motor learning objectives of Physical Education in many countries (European Commission/EACEA/Eurydice, 2013; Naul and Scheuer, 2020). Nevertheless, cross-national studies of motor competence are important in order to reveal the relevance of contextual influences and get helpful insights to develop tools for promoting motor proficiency in children. Such studies also give insights into similar or specific mechanisms and correlates of motor competence across diverse populations. So far, there are no studies comparing BMC levels across different countries. In the last years, a few studies that investigated and compared motor proficiency internationally using process- and product-oriented assessment tools (e.g., KTK, TGMD-2, TMC) found major differences in motor competence levels across geographical regions (Brian et al., 2018; Haga et al., 2018; Laukkanen et al., 2020). Even within Europe, children from Norway showed better results in fine and gross motor skills compared to children from Italy and Greece (Haga et al., 2018). Yet, many countries still lack established screenings of motor competence.

In a systematic review investigating correlates of gross motor competence, Barnett et al. (2016) found that sex strongly correlated with object control competencies. Girls from Belgium, the US, Australia and Finland scored lower in object control skills than boys (Barnett et al., 2016; Brian et al., 2018; Miller et al., 2019; Niemistö et al., 2019) but higher in locomotor skills than their male counterparts (Niemistö et al., 2019). In primary school children, higher age was associated with a higher level of motor competence (Barnett et al., 2016; Coppens et al., 2019). Body weight and motor competence showed relatively consistent inverse associations across different countries (Bardid et al., 2015; Coppens et al., 2019; Laukkanen et al., 2020).

Luz et al. (2019) proposed that differences in extracurricular sports participation were the reason for differences in motor competence levels between Portuguese and U.S. children. Participation in organized sports was associated with better object control and locomotion skills in Finland (Niemistö et al., 2019). Yet, the association between extracurricular PA (all regular PA outside of Physical Education) and motor competence has not been taken into account in cross-national studies. However, this association might impede the comparisons due to country-specific differences in extracurricular sport culture.

In order to provide a broad screening of BMC in Europe, we tested 6- to 8-year-old children in 10 European countries at the same time with the equal validated assessment tool. In this exploratory study, we also wanted to investigate the associations between BMC and possible individual determinants including age, sex, BMI and extracurricular PA among the subsamples and in the total sample.

## Materials and Methods

Twelve countries investigated BMC of children between 6 and 10 years in the Erasmus+ -project “*Basic Motor Competencies in Europe (BMC-EU) – Assessment and Promotion*” (590777-EPP-1-2017-1-DE-SPO-SCP). The project was led by teams from the University of Potsdam (Germany), the University of Luxembourg, and the University of Basel (Switzerland). Cross-sectional results of first and second grade children are presented here.

### Participants

Data was assessed in the third quarter of 2018 by 11 partner institutions in 10 countries (see Table 1). This study fully conforms to the Declaration of Helsinki. The partner institutions collected a data sample that was representative for the region they assessed. Prior to the testing, the participating institutions obtained ethical clearance for their respective sample. Parents gave written informed consent. Children assented orally.

**TABLE 1:** Descriptive statistics of age (mean ± *SD*, 95%*CI*) and BMI (mean, *SE*, 95%*CI*) and participation rates (% participating, 95%*CI*) in extracurricular physical activity, stratified by subsample site.

Subsample site	*N (of total sample)*	Girls	Age (in years)		Body mass index (kg/m^2^)^a^						Ball sport participation^b^		Individual sport participation^b^	
					
					Boys			Girls						
Salzburg (Austria)	207 (6%)	50%	7.47 ± 0.68	[7.38, 7.56]	16.21	0.22	[15.78, 16.64]	15.72	0.22	[15.29, 16.15]	59.9%	[52.2, 66.7]	88.9%	[84.2, 92.8]
Liège (Belgium)	299 (8%)	52%	6.42 ± 0.31	[6.39, 6.46]	15.62	0.15	[15.31, 15.92]	15.79	0.15	[15.50, 16.08]	20.4%	[15.7, 25.1]	64.6%	[59.0, 70.2]
Brno (Czech Republic)	255 (7%)	51%	6.94 ± 0.53	[6.88, 7.00]	15.82	0.19	[15.45, 16.19]	15.44	0.18	[15.08, 15.81]	43.5%	[37.1, 49.2]	72.9%	[67.7, 78.4]
Frankfurt/Main (Germany)	1503 (40%)	51%	6.75 ± 0.55	[6.72, 6.77]	16.27	0.09	[16.10, 16.45]	16.13	0.09	[15.96, 16.31]	20.5%	[18.6, 22.6]	30.7%	[28.4, 33.1]
Berlin (Germany)	565 (15%)	49%	6.68 ± 0.37	[6.65, 6.71]	15.31	0.11	[15.10, 15.52]	15.27	0.11	[15.06, 15.48]	37.8%	[33.8, 41.8]	52.2%	[48.1, 56.1]
Athens (Greece)	129 (3%)	50%	6.92 ± 0.55	[6.82, 7.01]	17.36	0.39	[16.60, 18.13]	17.29	0.39	[16.52, 18.06]	38.0%	[29.5, 47.3]	64.3%	[55.8, 72.1]
Luxembourg (Luxembourg)	275 (7%)	49%	7.09 ± 0.63	[7.01, 7.16]	15.55	0.18	[15.21, 15.90]	16.00	0.18	[15.64, 16.35]	32.7%	[27.6, 38.5]	62.2%	[56.2, 67.8]
Groningen (Netherlands)	154 (4%)	46%	7.09 ± 0.77	[6.97, 7.20]	16.11	0.23	[15.65, 16.57]	16.08	0.25	[15.58, 16.58]	22.1%	[15.7, 29.0]	85.7%	[79.9, 90.9]
Lisbon (Portugal)	114 (3%)	43%	7.46 ± 0.37	[7.40, 7.54]	17.02	0.34	[16.34, 17.70]	17.24	0.40	[16.46, 18.03]	43.9%	[34.5, 53.0]	64.9%	[56.1, 73.7]
Trnava (Slovakia)	105 (3%)	56%	6.70 ± 0.41	[6.63, 6.79]	16.26	0.40	[15.46, 17.06]	16.63	0.36	[15.92, 17.33]	61.0%	[51.9, 70.5]	77.1%	[68.6, 83.8]
Zurich (Switzerland)	152 (4%)	46%	7.56 ± 0.61	[7.46, 7.66]	15.69	0.16	[15.38, 16.00]	15.10	0.17	[14.76, 15.44]	29.6%	[22.7, 36.4]	61.2%	[53.3, 68.7]
Total sample	3758 (100%)	50%	6.86 ± 0.60	[6.84, 6.88]	16.02	0.05	[15.91, 16.12]	15.94	0.05	[15.84, 16.05]	30.6%	[29.1, 31.9]	52.0%	[50.3, 53.6]

*95% confidence intervals are added to test for differences between subsamples and total sample.*

*^a^BMI is adjusted for age.*

*^b^No participation = 0, participation = 1.*

*CI, confidence interval; N, sample size; SD, standard deviation; SE, standard error.*

The total sample with complete data (in all variables and covariates tested in this study) comprised *N* = 3758 first and second graders (50% girls) from 11 subsamples (see Table 1). For readability, the subsamples are named after the main city in the region of assessment: Salzburg (Austria), Liège (Belgium), Brno (Czech Republic), Frankfurt (Frankfurt/Main, Germany), Berlin (Germany), Athens (Greece), Luxembourg (Luxembourg), Groningen (Netherlands), Lisbon (Portugal), Trnava (Slovakia), Zurich (Switzerland).

Subsample sizes differed widely between *n* = 105 (Trnava) and *n* = 1503 (Frankfurt) (Table 1). The high number of participants in the latter is due to recruitment from a municipal screening.

### Instruments

#### Basic Motor Competencies

Basic motor competencies were assessed with the MOBAK-1-2 (Herrmann, 2018) for 6- to 8-year-old children with standardized equipment. The psychometric quality criteria for the MOBAK-1-2 have been confirmed several times in various validation studies via confirmatory factor analyses (Herrmann et al., 2015; Herrmann, 2018). The test instrument focuses on a total of eight items covering the two motor competence areas object movement (OM) and self-movement (SM) and is in line with several national Physical Education curricula (Greven and Letschert, 2006; Hessisches Kultusministerium, 2011; Österreichisches Bundesministerium für Bildung, Wissenschaft und Forschung, 2012; Herrmann et al., 2015; Landesinstitut für Schule und Medien Berlin-Brandenburg [LISUM], 2015; National Institute for Education [NIE], 2015; Deutschschweizer Erziehungsdirektoren-Konferenz, 2016; Ministère de l’Education nationale, de l’Enfance et de la Jeunesse [MENJE], 2017; Ministry of Education and Religious Affairs, 2017; Ministry of Education, Youth and Sports [MEYS], 2017; Administration générale de l’Enseignement [AGE] de la Fédération Wallonie-Bruxelles, 2020). We tested OM with the items throwing, catching, bouncing and dribbling and SM with the items balancing, rolling, jumping and running. For the six items bouncing, dribbling, balancing, rolling, jumping and running, each child performed two attempts (no trial run). For each turn, the test leader recorded whether the child passed or failed the attempt (failed attempt = 0 points; passed attempt = 1 point). The points from the two rounds were later summed per test item. For the test items throwing and catching, the children had six consecutive attempts each. The number of successful attempts was marked on the protocol and later transformed as follows: 0–2 successful attempts = 0 points, 3–4 successful attempts = 1 point, and 5–6 successful attempts = 2 points. Each competence area consisted of four test items, thus, allowing a maximum of eight points per competence area and a total of 16 points.

#### Age, Sex, and Body Mass Index

The test leader recorded the age (by month and year of birth) and sex of each child. Body height and weight for BMI calculation [mass(kg)/height(m^2^)] were measured using a scale (assessment in kg rounded to whole numbers) and a tape measure (assessment in cm rounded to whole numbers and later transferred to m).

#### Extracurricular Physical Activity

The test leader interviewed the children individually about their extracurricular PA (Do you participate in any kind of regular sport activities outside of school? What kind of sports do you participate in?) and recorded the type of sports on a standardized protocol using a dichotomous scale (0 = no extracurricular PA, 1 = extracurricular PA). Extracurricular PA was split posterior into two variables, namely ball sports (including *ball sports*) and individual sports (summarizing *racket sports, endurance-oriented activities* and *coordination-oriented activities*) according to the BMC areas OM and SM (Herrmann et al., 2017).

### Procedures

All tests were conducted during scheduled regular Physical Education classes. During the testing procedure, the classes were split into small groups of 4–5 students and assigned to specially certified and trained test leaders. The test leaders were sport scientists, future PE teachers or sport science students and experienced in conducting MOBAK-tests. Prior to the assessment, they participated in standardized trainer workshops using the same manual in all partner countries. The leaders guided their group through the test stations and assessed each child’s performance in a standardized protocol. The test leader explained the task with a given instructions sentence and gave one proficient demonstration for each test item of the MOBAK-1-2 (Herrmann et al., 2015). The order of the items was randomly chosen in each group. The oral interviews with the children were completed before or after measuring BMC. This procedure enabled an economical recording of one class within one school lesson.

### Data Analyses

All statistical analyses were performed using the software IBM SPSS Statistics 27 (IBM Corp., Armonk, NY, United States). We performed listwise deletion in order to obtain a total sample with complete data in OM, SM, age, BMI, sex and extracurricular PA variables (*N* = 4491 before data cleaning, *N* = 3758 after). We used Bootstrap 1000 with 95% confidence intervals (*CI*) in all analyses to compensate for the uneven subsample sizes.

Comparisons of anthropometric data and extracurricular PA among all subsamples were conducted using univariate analyses of covariance. BMI values were distinguished for girls and boys and adjusted for age. We calculated partial eta squared to analyze the effect size of the differences among the subsamples (0.01: small effect size; 0.06: medium effect size; 0.14 or higher: large effect size). 95%*CI* were considered in order to compare the subsamples with the total sample in anthropometric data and extracurricular PA participation (see Table 1).

Correlations of the BMC areas OM and SM with age were calculated using Pearson correlations including 95%*CI* (coefficient *r*; 0.1: small effect; 0.3: medium effect; 0.5 or higher: large effect; Cohen, 2013) (Table 2).

**TABLE 2:** Pearson correlations (*r*, 95%*CI*) between motor competence areas and age, stratified by subsample.

Subsample site	Age	
**Object movement**		
Salzburg	**0.40**	[0.27, 0.52]
Liège	**0.20**	[0.09, 0.29]
Brno	–0.02	[–0.15, 0.11]
Frankfurt	**0.19**	[0.14, 0.24]
Berlin	**0.09**	[0.01, 0.18]
Athens	**0.32**	[0.15, 0.48]
Luxembourg	**0.33**	[0.23, 0.42]
Groningen	**0.33**	[0.17, 0.48]
Lisbon	0.10	[–0.06, 0.23]
Trnava	0.05	[–0.13, 0.26]
Zurich	**0.41**	[0.26, 0.53]
Total	**0.27**	[0.24, 0.30]
**Self-movement**		
Salzburg	**0.21**	[0.09, 0.34]
Liège	**0.18**	[0.07, 0.28]
Brno	**−−0.14**	[–0.26, –0.02]
Frankfurt	–0.01	[–0.06, 0.05]
Berlin	**0.10**	[0.02, 0.18]
Athens	0.15	[–0.04, 0.31]
Luxembourg	–0.07	[–0.20, 0.07]
Groningen	**0.25**	[0.12, 0.39]
Lisbon	0.09	[–0.11, 0.35]
Trnava	–0.03	[–0.18, 0.13]
Zurich	**0.40**	[0.25, 0.52]
Total sample	**0.13**	[0.10, 0.16]

*95% confidence intervals are added to test for differences between subsamples and total sample. Significant coefficients are bold.*

Marginal estimates are displayed for BMC values of boys and girls per sample site including Cohen’s *d* in order to investigate differences in BMC levels between boys and girls per subsample (0.2: small effect; 0.5: medium effect; 0.8: large effect; Table 3).

**TABLE 3:** Marginal estimates of basic motor competencies (mean, *SE*, 95%*CI*) per competence area and sex, stratified by subsample site and including Cohen’s *d* for effect size of differences between boys and girls.

Subsample site	Boys			Girls			Cohen’s *d*
**Object movement**							
Salzburg	6.18	0.16	[5.87, 6.50]	4.99	0.16	[4.68, 5.30]	0.75
Liège	4.60	0.15	[4.30, 4.90]	3.44	0.15	[3.15, 3.72]	0.64
Brno	5.43	0.15	[5.14, 5.72]	4.55	0.14	[4.27, 4.84]	0.53
Frankfurt	4.67	0.07	[4.54, 4.81]	3.07	0.07	[2.94, 3.20]	0.85
Berlin	5.18	0.11	[4.98, 5.39]	3.88	0.11	[3.67, 4.09]	0.73
Athens	5.18	0.26	[4.66, 5.71]	3.39	0.27	[2.87, 3.92]	0.85
Luxembourg	4.99	0.17	[4.67, 5.32]	3.72	0.17	[3.38, 4.05]	0.65
Groningen	4.84	0.20	[4.44, 5.25]	3.96	0.22	[3.52, 4.39]	0.48
Lisbon	5.98	0.21	[5.57, 6.40]	3.41	0.24	[2.93, 3.89]	1.52
Trnava	4.00	0.23	[3.54, 4.46]	2.92	0.20	[2.51, 3.32]	0.69
Zurich	6.04	0.19	[5.65, 6.42]	4.67	0.21	[4.26, 5.09]	0.78
Total sample	5.02	0.04	[4.93, 5.10]	3.58	0.04	[3.50, 3.67]	0.75
**Self-movement**							
Salzburg	6.02	0.16	[5.71, 6.33]	6.19	0.16	[5.88, 6.50]	–0.11
Liège	4.87	0.14	[4.58, 5.15]	5.29	0.14	[5.02, 5.57]	–0.25
Brno	5.67	0.15	[5.37, 5.97]	6.14	0.15	[5.84, 6.43]	–0.27
Frankfurt	3.86	0.08	[3.71, 4.02]	4.00	0.08	[3.85, 4.15]	–0.06
Berlin	5.32	0.09	[5.14, 5.50]	5.70	0.09	[5.52, 5.89]	–0.25
Athens	3.92	0.23	[3.46, 4.39]	4.63	0.24	[4.16, 5.09]	–0.38
Luxembourg	5.38	0.16	[5.07, 5.68]	5.69	0.16	[5.37, 6.00]	–0.17
Groningen	5.88	0.19	[5.50, 6.26]	6.31	0.21	[5.90, 6.72]	–0.25
Lisbon	4.94	0.22	[4.50, 5.37]	4.24	0.25	[3.74, 4.75]	0.39
Trnava	5.57	0.20	[5.18, 5.96]	5.29	0.17	[4.94, 5.63]	0.21
Zurich	5.73	0.19	[5.36, 6.10]	6.27	0.20	[5.87, 6.67]	–0.32
Total sample	4.77	0.05	[4.68, 4.86]	4.99	0.05	[4.89, 5.08]	–0.11

*95% confidence intervals are added to test for differences between subsamples and total sample. Significant coefficients are bold.*

*CI, confidence interval; SE, standard error.*

Table 4 shows adjusted BMC values per competence area for each subsample and the total sample as well as intercorrelations between OM and SM for each subsample (Table 4, adjusted for age and sex). Univariate analyses of covariance allowed for overall in between subsample comparison of BMC values and 95%*CI* for comparisons of subsamples with the total sample. We calculated partial Pearson correlations of both BMC areas with the correlates BMI, individual sports and ball sports. Correlations included adjustments for age and sex in order to account for the variance of these covariates (Table 4).

**TABLE 4:** Sum values of basic motor competencies (mean, *SE*, 95%*CI*) per competence area, Partial Pearson correlations (*r*, 95%*CI*) between competence areas per subsample and between motor competence areas and BMI and extracurricular physical activity participation, stratified by sample site.

Subsample site	Basic motor competencies			Correlation of object movement and self-movement		Body mass index		Ball sport participation		Individual sport participation	
**Object movement**											
Salzburg	5.18	0.13	[4.95, 5.40]	**0**.**23**	[0.09, 0.36]	–0.03	[–0.15, 0.09]	**0**.**15**	[0.00, 0.28]	0.06	[–0.09, 0.21]
Liège	4.31	0.11	[4.11, 4.53]	**0**.**24**	[0.14, 0.35]	0.01	[–0.10, 0.11]	**0**.**21**	[0.09, 0.31]	–0.02	[–0.12, 0.10]
Brno	4.94	0.11	[4.72, 5.15]	**0**.**37**	[0.26, 0.46]	0.04	[–0.09, 0.18]	**0**.**30**	[0.19, 0.42]	–0.06	[–0.18, 0.06]
Frankfurt	3.94	0.05	[3.84, 4.04]	**0**.**34**	[0.29, 0.38]	–0.03	[–0.07, 0.02]	**0**.**23**	[0.18, 0.27]	**0.06**	[0.01, 0.11]
Berlin	4.65	0.08	[4.51, 4.80]	**0**.**26**	[0.19, 0.34]	**0.11**	[0.02, 0.19]	**0**.**11**	[0.02, 0.18]	0.03	[–0.06, 0.11]
Athens	4.25	0.16	[3.89, 4.60]	**0**.**35**	[0.20, 0.49]	–0.03	[–0.18, 0.13]	**0**.**40**	[0.25, 0.54]	–0.04	[–0.22, 0.12]
Luxembourg	4.20	0.11	[3.97, 4.42]	**0**.**31**	[0.20, 0.41]	–0.05	[–0.16, 0.09]	0.09	[–0.02, 0.20]	**0.13**	[0.01, 0.25]
Groningen	4.23	0.15	[3.96, 4.48]	**0**.**22**	[0.07, 0.36]	–0.06	[–0.19, 0.07]	**0**.**19**	[0.06, 0.33]	–0.09	[–0.24, 0.08]
Lisbon	4.38	0.17	[4.02, 4.72]	**0**.**27**	[0.10, 0.43]	–0.15	[–0.33, 0.03]	0.07	[–0.11, 0.27]	0.00	[–0.20, 0.18]
Trnava	3.58	0.18	[3.26, 3.88]	0.16	[–0.10, 0.38]	0.00	[–0.14, 0.19]	-0.07	[–0.27, 0.13]	**−−0.36**	[–0.54, –0.13]
Zurich	4.89	0.15	[4.60, 5.17]	**0**.**18**	[0.01, 0.34]	–0.16	[–0.34, 0.04]	0.10	[–0.07, 0.26]	**0.18**	[0.04, 0.33]
Total	4.30	0.03	[4.23, 4.36]	**0**.**34**	[0.31, 0.37]	**−−0.04**	[–0.07, –0.01]	**0**.**21**	[0.18, 0.24]	**0.08**	[0.05, 0.12]
**Self-movement**											
Salzburg	5.99	0.13	[5.76, 6.21]			**−−0.26**	[–0.40, –0.11]	0.12	[–0.03, 0.26]	0.12	[–0.03, 0.25]
Liège	5.17	0.11	[4.97, 5.36]			**−−0.13**	[–0.23, –0.02]	0.09	[–0.02, 0.19]	**0.12**	[0.02, 0.24]
Brno	5.89	0.12	[5.67, 6.10]			–0.04	[–0.17, 0.09]	**0**.**13**	[0.00, 0.25]	–0.04	[–0.17, 0.09]
Frankfurt	3.95	0.05	[3.85, 4.06]			**−−0.15**	[–0.20, –0.11]	0.09	[0.04, 0.14]	**0.15**	[0.10, 0.20]
Berlin	5.55	0.08	[5.41, 5.67]			**−−0.08**	[–0.16, –0.01]	0.07	[–0.02, 0.16]	**0.09**	[0.01, 0.18]
Athens	4.26	0.16	[3.93, 4.61]			**−−0.23**	[–0.39, –0.06]	**0**.**22**	[0.06, 0.37]	–0.06	[–0.21, 0.11]
Luxembourg	5.49	0.11	[5.26, 5.72]			**−−0.14**	[–0.28, 0.00]	0.03	[–0.08, 0.14]	0.09	[–0.03, 0.22]
Groningen	6.05	0.15	[5.77, 6.31]			**−−0.25**	[–0.39, –0.09]	0.02	[–0.13, 0.17]	0.02	[–0.15, 0.21]
Lisbon	4.54	0.18	[4.20, 4.87]			**−−0.21**	[–0.41, –0.03]	0.10	[–0.07, 0.27]	0.01	[–0.19, 0.18]
Trnava	5.42	0.18	[5.17, 5.69]			0.05	[–0.09, 0.23]	**0**.**32**	[0.14, 0.49]	**0.30**	[0.11, 0.50]
Zurich	5.86	0.16	[5.57, 6.15]			**−−0.18**	[–0.33, –0.02]	**0**.**17**	[0.02, 0.32]	0.07	[–0.10, 0.25]
Total	4.88	0.03	[4.81, 4.94]			**−−0.19**	[–0.22, –0.16]	**0**.**15**	[0.12, 0.18]	**0.23**	[0.20, 0.27]

*All values are adjusted for age and sex.*

*95% confidence intervals are added to test for differences between subsamples and total sample. Significant coefficients are bold.*

*CI, confidence interval; SE, standard error.*

## Results

### Sample Characteristics

Nine of the 11 *ad hoc* subsamples consisted of 100–300 children with five subsamples consisting of fewer than 200 children. Distribution across sex was well-balanced in the subsamples and the total sample (Table 1).

The individual factors varied between the subsamples. Differences were found in age [*F*(10,3746) = 112.66, *p* < 0.001, range: 6.42 (Liège) – 7.56 (Zurich), η^2^ = 0.231], participation in ball sports [*F*(10,3745) = 32.01, *p* < 0.001, range 20.4% (Liège) – 61.0% (Trnava), η^2^ = 0.079] and participation in individual sports [*F*(10, 3745) = 66.62, *p* < 0.001, range: 30.7% (Frankfurt) – 88.9% (Salzburg), η^2^ = 0.151]. Overall participation in extracurricular PA differed internationally with Frankfurt having the lowest and Salzburg having the highest participation rate. In BMI, we found differences between the subsamples for the boys [*F*(10,1860) = 8.58, *p* < 0.001, range: 15.31 (Berlin) – 17.36 (Athens), η^2^ = 0.044] as well as for the girls [*F*(10, 1874) = 9.38, *p* < 0.001, range: 15.10 (Zurich) – 17.29 (Athens), η^2^ = 0.048].

### Levels of Basic Motor Competencies

As Table 4 shows, the mean value of the total sample in OM was 4.30 and was surpassed by six samples. Five of these six samples as well as three others also exceeded the mean value of the total sample in SM which was 4.88. The BMC level controlled for sex and age especially differed between the subsamples regarding SM [OM: *F*(10, 3745) = 18.74, *p* < 0.001, range: 3.58 (Trnava) – 5.18 (Salzburg), η^2^ = 0.048; SM: *F*(10, 3745) = 73.10, *p* < 0.001, range: 3.95 (Frankfurt) – 6.05 (Groningen), η^2^ = 0.163]. Intercorrelations were significant in the total sample (*r* = 0.34) and in all subsamples except the Trnava subsample (*r* = 0.16). Differences in intercorrelations between OM and SM did not vary substantially between the subsamples (see Table 4).

### Correlates of Levels of Basic Motor Competencies

#### Age, Sex, and Body Mass Index

In the total sample, boys clearly performed better in OM [*t*(3756) = 23.09, *p* < 0.001, *d* = 0.75] while girls only slightly performed better in SM [*t*(3756) = –3.24, *p* < 0.01, *d* = −0.11] (Table 3). Consequently, in every subsample, boys were significantly better in OM with medium to high effect sizes. In SM, the effects were small with girls performing significantly better than boys in four subsamples and significantly worse than boys in one subsample (Lisbon).

Correlations between BMC values and age showed that older children performed better in both OM and SM in all samples including the total sample (Table 2). In OM, there was a moderate effect for age (*r* = 0.27) whereas in SM the effect was small (*r* = 0.13). The strongest association with age was apparent in the samples from Salzburg and Zurich for OM and in the Zurich sample for SM. Only the subsample from Brno showed a small negative correlation with age in SM.

The correlation between BMI and OM showed a negative tendency in the total sample. Only the subsample from Berlin had a significant small positive correlation. Besides these findings, there were no significant correlations between OM and BMI. In SM, the total sample as well as all subsamples except the Brno and Trnava samples showed a significant small negative correlation (Table 4).

#### Extracurricular Physical Activity

Children’s participation in extracurricular PA was linked to higher results in both competence areas. These results were consistent in the majority of the subsamples as well as the total sample. Stronger associations were found between the specific type of PA and motor competence (Table 4). Even though many samples did not show significant correlations between the motor competence areas and extracurricular PA, at least the tendency was the same in almost all subsamples.

Children who participated in ball sports showed small to moderate correlations with OM in seven samples and in the total sample whereas the correlation between SM and participation in ball sports was also small to moderate in four samples and small in the total sample.

Correlations between OM and individual sports varied from a moderate negative correlation (Trnava) to a small positive correlation (Zurich) while the total sample showed no correlation. In total, only three subsamples had significant *r*-values above or below zero. This inconsistency shows that participation in individual sports was not associated with performance in OM tasks in general. Participation in individual sports correlated significantly with SM in the total sample. Three subsamples had significant small to moderate correlations. Overall, correlations in the total sample were strongest between OM and ball sports as well as between SM and individual sports (Table 4).

## Discussion

The purpose of our study was to analyze levels of and associations with BMC in 6- to 8-year-old primary school children in 10 European countries. Our study is the first to provide an overview of BMC across different regions using the same assessment tool, namely the MOBAK-1-2 test. The findings show strong variation in BMC and, therefore, in achieving a key learning goal of Physical Education, but similar associations with determinants including age, sex, BMI, and extracurricular PA across the subsamples. These results indicate that children’s dispositions to actively participate in sports depend on where they live.

### Levels of Basic Motor Competencies

Overall, children from all subsamples scored moderately in both OM and SM. We found significant cross-national differences in BMC levels across the countries and regions we assessed. The associations between OM and SM tasks were positive throughout. Especially in SM tasks the results varied significantly between the subsamples. These results are in line with a recent cross-European study in which the geographic region explained 19% of variance in motor competence (Laukkanen et al., 2020). That study compared the levels of motor competence across three different regions in Europe (northern, central and southern Europe) and found considerable differences in 6–9-year old children’s gross motor coordination and body control. The authors assumed that those differences were due to varying developmental rates of motor competence in children as well as individual and environmental factors including government strategies and active play in childhood. Nevertheless, the researchers were unable to fully explain the reasons for the variation. The authors of another study comparing fine and gross motor competence levels of Greek, Italian and Norwegian children assumed that differences in motor competence in their study were unlikely due to anthropometric reasons. Instead, they ascribed these differences to country-specific differences in Physical Education objectives and frameworks in preschool and primary school (Haga et al., 2018). The exploratory structure of our study does not allow us to fully explain the differences between BMC levels across the subsamples. We assume that determining factors like country-specific variations in content and setting of Physical Education and in leisure time activities led to these results. Children in first and second grade have only been experiencing Physical Education for a short time. As a result, it is possible that they are not yet familiar with some specific movements or content of Physical Education. Furthermore, not all countries offer Physical Education in kindergarten already, and the duration of kindergarten also varies. As the MOBAK-1-2 is strongly aligned with the curricular content, differences in BMC could emerge. Another aspect could be the importance and extent of sports in the family. To compensate for such disparities and to avoid overlooking children with low motor competence, regular BMC assessments throughout childhood as well as specific interventions to foster BMC would be beneficial. On the school level, support tools and educational materials for teachers on how to improve BMC in their classes may enable children to achieve a basic level of motor competence through Physical Education (Scheuer and Heck, 2020).

### Correlates of Basic Motor Competencies

Overall, boys of all samples in our study performed better in OM tasks than girls while girls tended to score higher in SM tasks than boys. This corresponds with preceding studies assessing BMC (Herrmann, 2018) as well as research about general motor competence (Barnett et al., 2016; Niemistö et al., 2019; Pill and Harvey, 2019) and met our expectation. Surprisingly, one subsample (Lisbon) showed an opposite result with boys scoring significantly higher in SM than girls. This finding was also reported by a study on fundamental movement skills where boys outperformed girls in locomotion skills, even though this assessment focused on the quality of the movement and not on the successful mastery of a task (Jiménez-Díaz et al., 2015). These differences between the sexes can be due to the curricula, the sports culture in the region or country or the specific regional and/or socioeconomic sample, as several studies have suggested (Brian et al., 2018; Haga et al., 2018; Luz et al., 2019). However, given the low *ad hoc* conducted sample size in the Lisbon subsample, these interpretations remain unclear.

Consistent with the literature (Herrmann, 2018), this study showed that age is positively associated with BMC. Older children have higher BMC scores than younger children in the same age group.

Object movement tasks appear to be relatively non-sensitive to body size and proportion. We found no correlation between BMI and OM in the total sample or in the subsamples with one exception. These results match previous BMC research (Herrmann, 2018). There seem to be other factors that influence OM more than BMI because in previous studies BMI also did not appear to impact OM. Only the Berlin subsample showed a small positive correlation between BMI and OM, which might be attributed to the lowest mean BMI and therefore small variance in this subsample.

For SM, the results showed that children with a lower BMI scored higher in BMC. These findings are consistent with previous BMC research as well as research on motor competence showing strong scientific proof of an inverse association between body weight and motor competence which even increases with age (Bardid et al., 2015; Cattuzzo et al., 2016; Herrmann and Seelig, 2017; Strotmeyer et al., 2020). A longitudinal study showed negative associations between gross motor competence and BMI at baseline as well as in the 2-year follow up. Additionally, worse performance in gross motor competence predicted an increase in BMI and vice versa (D’Hondt et al., 2014). Another study with 6- to 10-year old children found that the current BMI was a significant predictor of future performances in motor competence (D’Hondt et al., 2013). These results highlight the importance of early detection of children’s weight status (focusing on high BMI levels) and motor competence levels (focusing on poor motor competence levels) because of their intertwining relationship over time.

In our study, participation rates in extracurricular PA varied widely from rather low to extremely high. Almost one third of all children participated in ball sports while more than half of the children were active in individual sports outside of school. Some subsamples were noticeable for their exceptional high rates of participation in individual sports (e.g., Salzburg and Groningen with both above 85%). These children also showed the highest values in SM. This could explain the left-skewed distribution of SM scores in the total sample with 60.7% of the children scoring above average in SM. Of the children with above average SM scores, 60.3% participated in individual sports outside of school and 35% in ball sports. The subsample with the lowest participation rate in individual sports (Frankfurt) likewise reached the lowest BMC scores in SM. Interestingly, there was only a small correlation between SM and individual sport participation in this subsample and no correlation in the Groningen and Salzburg subsamples. The subsamples with participation rates in extracurricular ball sport activities below 30% did not show lower OM competencies than the other subsamples. Of the two subsamples with more than 59% of the children participating in ball sports, one showed the highest (Salzburg) and the other one the lowest (Trnava) BMC value in OM. These surprising results could be due to the fact that the Trnava sample consisted of more girls than the other subsamples (and they scored lowest in OM).

Even though differences between extracurricular PA participation were apparent, their relation to BMC was similar across the majority of the samples. Overall, our study confirmed previous findings about the positive relationship of participation in PA outside of school and higher motor competence levels (Drenowatz and Greier, 2019; Schembri et al., 2019; Niemistö et al., 2020) which was particularly visible in specific types of PA and motor competence. Children participating in ball sports outside of school showed higher success rates in OM tasks than children who did not participate in such activities. Likewise, children participating in individual sports showed higher success rates in SM tasks than their non-participating counterparts.

Schembri et al. (2019) showed that primary school children who were active more than 120 min per week were more likely to have a higher level of motor competence than less active children. This finding supports the effort in fostering participation of children in extracurricular sports activities (Fairclough and Stratton, 2006; Meyer et al., 2013; Hollis et al., 2016; Kühnis et al., 2017; Tanaka et al., 2018).

Among Flemish children between 6 and 11 years, boys and girls participating in sport activities and sport clubs had more likely a high socioeconomic status than children who were not participating in those activities (Vandendriessche et al., 2012). Furthermore, the hours spent participating in sport were a significant covariate for motor coordination variables like jumping sideways or balancing backwards on a balance beam. A longitudinal study showed that consistent sports club participation over 3 years was associated with better motor coordination levels than no sports club participation (Vandorpe et al., 2012). Additionally, the motor coordination level predicted sports club participation 2 years later. Drenowatz and Greier (2019) confirmed this finding for motor competence as a predictor variable.

With the importance of frequent and extensive PA and socioeconomic status as an important influence factor for participation in extracurricular PA, it is important that extracurricular PA opportunities are made available to everyone with a focus on lower income families.

### Strengths and Limitations

The main strength of the study lies in the widespread simultaneous assessment of BMC across 10 European countries using the same standardized procedures, equipment and test instrument. The test instrument, MOBAK-1-2, is aligned with key learning goals of Physical Education in these countries. All data was managed and analyzed centrally. The MOBAK test instrument covers two main areas addressing both object movement and self-movement competencies, and, therefore, offers a broad insight into children’s motor competence levels. The total sample size is remarkable and provides a deeper understanding of motor competence levels and associations in different regions in Europe. The assessed data allows for further country-specific research and longitudinal screenings.

Despite these strengths, limitations should be considered. Due to the lack of representative data for the different countries, direct comparisons between the countries are problematic and sometimes limited to specific circumstances of the subsamples. Assessment fidelity was not controlled for, even though the use of a standardized test manual and practical trainings for the test leaders were implemented to ensure a high level of standardization. Differences in anthropometric data between the subsamples could be the result of contextual influences of different regions. Therefore, adjusted statistics were used to reduce the impact of differences in anthropometric data. Future studies with more representative samples and higher sample sizes per country are recommended. To reveal more about the causes of the differences between the subsamples, more detailed information on the sociocultural background of individuals, as well as information at the national level, should be considered. The subsamples are, however, still representative for their specific region of assessment and offer reference points for further investigations. Information about PA beyond self-reported extracurricular PA including physical fitness levels as well as other measures related to BMC were not obtained. A more thorough assessment of such variables would foster the understanding of BMC and important correlates even more. The use of validated scales and stadiometers across all samples would allow more precise assessments of children’s height and weight. More specific measurements of children’s body composition (e.g., bioelectrical impedance analyses) would allow a more accurate statement than the BMI value (Pecoraro et al., 2003; Kriemler et al., 2009). Lastly, only a small part of motor competence was examined. Other studies, like Scheuer et al. (2019a), tested further aspects of motor competence (e.g., moving in water or object-locomotion) and were, therefore, able to display a broader picture of motor competence levels of children than our study.

## Conclusion

The study’s sample suggests that BMC levels in primary school children in many European countries vary strongly depending on the country and region they are living in. The vast amount of assimilated data contributes to a deeper understanding of the current status of BMC in children and lays the foundation for further research and longitudinal monitoring on a national or international level. For some countries, this study was the first assessment of children’s BMC levels. The differences in BMC levels may be attributed to specific cultural conditions and educational settings like country-specific Physical Education frameworks and should be explored in more detail. Furthermore, education and health policies to enhance BMC development as well as regular monitoring of BMC should be established.

Confirming previous research, associations between BMC and correlates like age, sex, BMI and extracurricular PA were similar across all subsamples. These results indicate that the MOBAK instruments are feasible for screening BMC in many European countries regardless of the cultural context. The strong associations between BMC levels and extracurricular PA in all subsamples highlight the importance of fostering the offer of and access to PA outside of Physical Education.

## Data Availability Statement

The datasets used and analyzed during the current study are available from the corresponding author on reasonable request.

## Ethics Statement

The studies involving human participants were reviewed and approved by the following ethics committees: research ethics committee from Masaryk University (Brno, Czechia); scientific committee from the School of Physical Education and Sport Science of the National and Kapodistrian University of Athens (Athens, Greece); ethics committee from Paris Lodron University of Salzburg (Salzburg, Austria); ethical advise committee from the Hanze University of Applied Sciences (Groningen, Netherlands); ethics committee from the University Hospital of Liège (Liège, Belgium); ethics review panel from the University of Luxembourg (Luxembourg, Luxembourg); ethics committee from University of Potsdam (Potsdam, Germany); and ethics committee from Northwest and Central Switzerland (Zurich, Switzerland). Written informed consent to participate in this study was provided by the participants’ legal guardian/next of kin.

## Author Contributions

MW was responsible for study management and analyzed the data and drafted the manuscript. JS was responsible for overall organization of the study and revised the manuscript. CHer was responsible for the overall conception and design of this study, contributed to data analysis and interpretation of results and revised the manuscript. Other authors were responsible for data assessment and management in their regions and country-specific additions to the study. All authors provided critical feedback to the manuscript, have read and approved the final version of the manuscript, and agreed with the order of presentation of the authors.

## Conflict of Interest

The authors declare that the research was conducted in the absence of any commercial or financial relationships that could be construed as a potential conflict of interest.

## Publisher’s Note

All claims expressed in this article are solely those of the authors and do not necessarily represent those of their affiliated organizations, or those of the publisher, the editors and the reviewers. Any product that may be evaluated in this article, or claim that may be made by its manufacturer, is not guaranteed or endorsed by the publisher.

## Abbreviations

BMC: basic motor competencies
BMI: body mass index
OM: object movement
PA: physical activity
SM: self-movement.
